# Evolutionarily Conserved Network Properties of Intrinsically Disordered Proteins

**DOI:** 10.1371/journal.pone.0126729

**Published:** 2015-05-14

**Authors:** Nivedita Rangarajan, Prakash Kulkarni, Sridhar Hannenhalli

**Affiliations:** 1 Indian Institute of Science Education and Research, Pune, India; 2 Institute for Bioscience & Biotechnology Research, University of Maryland, Rockville, Maryland, United States of America; 3 Center for Bioinformatics and Computational Biology, Department of Cell Biology and Molecular Genetics, University of Maryland, College Park, Maryland, United States of America; Universita' di Padova, ITALY

## Abstract

**Background:**

Intrinsically disordered proteins (IDPs) lack a stable tertiary structure in isolation. Remarkably, however, a substantial portion of IDPs undergo disorder-to-order transitions upon binding to their cognate partners. Structural flexibility and binding plasticity enable IDPs to interact with a broad range of partners. However, the broader network properties that could provide additional insights into the functional role of IDPs are not known.

**Results:**

Here, we report the first comprehensive survey of network properties of IDP-induced sub-networks in multiple species from yeast to human. Our results show that IDPs exhibit greater-than-expected modularity and are connected to the rest of the protein interaction network (PIN) via proteins that exhibit the highest betweenness centrality and connect to fewer-than-expected IDP communities, suggesting that they form critical communication links from IDP modules to the rest of the PIN. Moreover, we found that IDPs are enriched at the top level of regulatory hierarchy.

**Conclusion:**

Overall, our analyses reveal coherent and remarkably conserved IDP-centric network properties, namely, modularity in IDP-induced network and a layer of critical nodes connecting IDPs with the rest of the PIN.

## Introduction

Complex biological processes that govern cellular functions cannot be entirely understood at the level of individual genes and proteins. Instead, a systems-level investigation that includes interactions among genes and proteins is essential to gain a comprehensive appreciation of how complex biological systems function and interact with their environment [1]. Recent availability of large-scale interaction data as well as other high-throughput datasets [2] have fuelled systems-level approaches that apply network theory to gain insights into how biological systems are organized to facilitate robust and rapid information processing [3].

Previous investigations have shown that biological networks such as protein interaction networks (PINs) adopt a scale-free architecture, wherein relatively few nodes have high connectivity (hubs) and the vast majority of nodes have a diminishingly low degree of interaction [4]. Furthermore, beyond the analysis of hubs and degree distribution, several groups have elucidated other network properties such as clustering coefficient, shortest path length, betweenness centrality, etc. [5,6,7,8].

Our focus here is on a very important class of proteins – intrinsically disordered proteins (IDPs) [9]. IDPs lack a stable tertiary structure in isolation [10,11], but typically possess the remarkable ability to undergo disorder-to-order transitions upon binding to their biological target, a phenomenon referred to as coupled folding and binding [12]. Structural flexibility and plasticity represent a major functional advantage for the IDPs enabling them to interact with a broad range of binding partners [10,11]. At the same time, because of their inherent flexibility, IDPs are prone to initiate promiscuous molecular interactions when overexpressed, resulting in toxicity/pathology. Indeed, IDPs are frequently overexpressed in several pathological states [13] and not surprisingly, IDPs tend to be dosage-sensitive [14]. Consequently, their expression is tightly regulated from transcript synthesis to protein degradation [15].

Beyond their tendency to have a greater number of interactions, other distinguishing and evolutionarily conserved characteristics of IDP-induced networks are not known. In the present study, based on the available PINs as well as protein disorder annotation in four broadly distributed species, namely, *Saccharomyces cerevisiae* (yeast), *Drosophila melanogaster* (fly), *Mus musculus* (mouse) and *Homo sapiens* (human), we have analyzed, for the first time, a comprehensive set of network properties (Table 1) of the sub-networks induced by the IDPs (*NetIDP*). Previous investigations of regulatory networks have proposed a hierarchical structure, where certain transcription factors assume the role of master regulators and affect gene expression at the bottom level via a layer of “middle level” managers [16]. Considering the central role of IDPs, we analyzed various properties of immediate neighbors of IDPs to assess their roles as mediators. Broadly, our results suggest that IDPs are enriched at higher levels of regulatory hierarchy and exhibit relatively high modularity and low efficiency. IDPs are connected to the rest of the network *via* a middle layer of non-IDP (structured) proteins that critically mediate, as evidenced by high betweenness centrality, the communication between the IDPs and the rest of the PIN.

**Table 1:** Network properties investigated.

**Network Density:** The ratio of the numbers of edges in the network and the total possible edges.
**Clustering Coefficient:** *N*_*u*_ is the set of neighbors of a node *u*. The local clustering coefficient for node *u* is the ratio of number of edges between nodes in *N*_*u*_ and the total possible number of edges between nodes in *N*_*u*_, that is, *N*_*u*_-choose-2. The clustering coefficient of a network is the average local clustering coefficient of all nodes.
**Transitivity Coefficient:** It is related to the clustering coefficient, and is the ratio of the number of closed triplets (*u* connected to *v* connected to *w* connected to *v*) to the number of connected triplets (at least 2 of the 3 edges present) of nodes in the network.
**Rich Club Coefficient:** It is related to the network density but only includes the nodes with degree above a threshold *k*. That is, for a given *k*, the rich club coefficient is the ratio of the number of actual to the number of potential edges between nodes with degree greater than *k* [19]. The normalized rich club coefficient was calculated, with respect to a degree-preserving randomized network. Thus, a normalized rich club coefficient >1 indicates that the network has a rich club structure, whereas a value <1 indicates otherwise.
**Characteristic Path Length:** Shortest path length between a pair of nodes is the number of edges in the shortest path between the two nodes. Characteristic path length is the average pairwise shortest path lengths between all pairs of nodes. The standard convention to estimate characteristic path length in a disconnected graph ignores the cross-component node pairs [52].
**Efficiency:** *T*he global efficiency is defined as the harmonic mean of all pairwise shortest path lengths [21]. It measures the efficiency with which nodes in a network can exchange information with each other. Efficiency is not monotonically related to characteristic path length, and for a fixed characteristic path length, efficiency attains a maximum value when the pair-wise shortest path lengths have low variability.
**Modularity:** The network is divided into ‘communities’. Each community consists of disjoint set of nodes that are linked more with other nodes in the same community, compared to the nodes of another community [20]. This is done such that the number of edges between different communities is minimized, and the number of nodes within a community is maximized. Given a community structure, modularity is the fraction of the edges that connect two nodes within the same community minus the expected fraction if edges were distributed at random [53]. Formally, modularity is computed as: *Q* = ∑_*u*∈*M*_[*e*_*uu*_ – (∑_*v*∈*M*_ *e*_*uv*_)^2^], where M is the set of non-overlapping modules that the whole network is sub-divided into, and *e*_*uv*_ is the proportion of all the links that connect nodes in module *u* with those in *v*.
**Betweenness Centrality:** For node *u*, betweenness centrality is the fraction of all shortest paths (between all node pairs) that pass through *u*. Thus, a higher value of betweenness centrality indicates a greater participation of that particular node in the shortest paths between any two nodes of the network [54].
**IDP Identification.** MobiDB database [45] integrates multiple sources to provide a consensus annotation of disorder for each residue in a protein. We defined disordered regions as stretches of at least 30 disordered residues. As described previously, the overall disorder score of a protein is the fraction of protein sequence covered by disordered regions [9,46]. A protein was designated as an IDP if the disorder score was above a certain threshold. We used two thresholds, namely, 50% and 70% disorder.

## Results

### Overview

Our overall goal was to assess whether IDP-induced sub-networks exhibit unexpected topological properties. To define IDPs, we used annotations provided in MobiDB database (see Materials & Methods). The disorder score for a protein sequence is calculated as the fraction of protein sequence covered by disordered regions. For all analyses, we used two stringent thresholds for the disorder score– 50% and 70%. Independently in all four species, we estimated a broad array of network properties (Table 1) of sub-networks induced by IDPs (NetIDP) and the entire PIN (NetAll). We assessed the statistical significance of NetIDP’s topological properties based on the distribution obtained from 300 degree-preserving randomizations of NetIDP. With minor exceptions, all cases that were deemed significant using our analytical approach (see Materials & Methods), were also significant using a direct empirical estimate.

Besides applications to four species and using two disorder thresholds, to further ascertain robustness of our results, we repeated our analyses using multiple sources for protein networks (see Materials & Methods): (1) STRING Physical Interaction: For human, mouse, fly, and yeast, we used the physical interactions from STRING database [17]. (2) BIOGRID: For human and yeast we used BIOGRID database [18]. For mouse and fly, BIOGRID does not contain sufficient data for statistical analyses. (3) STRING integrated (functional) network: For the four species, we also used the integrated STRING database.

All network properties for all four species at all disorder stringencies and all network databases are listed in Table A in S1 File. The statistical significance of NetIDP properties are included in Tables B and C in S1 File. Further details of various aspects of our pipeline are provided in Materials and Methods section. Next we present our analyses of various network properties of NetIDP. Unless otherwise mentioned our results refer to those based on STRING physical interaction at 70% disorder threshold.

### Network Density

S1 Fig shows the degree distribution of IDPs (at 70% threshold) and all nodes for the 4 species, based on STRING physical interaction data. The overall degree distributions do not reveal an obvious difference between IDPs and non-IDPs. However, by and large, with very few exceptions, in four species, for two thresholds, and for three different network data sources, the network density of NetIDP is greater than that of NetAll, (Table A in S1 File). When we explicitly assessed the enrichment of hubs (defined as nodes with degree > mean + standard deviation of all degrees in NetAll) we did not see a robust and consistent signal for enrichment of hubs among IDPs as observed previously [9].

### Clustering, Transitivity and Rich club Coefficient

The two related measures, clustering and transitivity coefficient (also called ‘global’ clustering coefficient) capture local clustering of nodes; while clustering coefficient measures the extent to which neighbors of a node are connected to each other, the transitivity coefficient quantifies whether ‘the friend of a friend is a friend’. Given a slightly greater edge density in NetIDP relative to NetAll, probabilistically, one would expect a greater clustering and transitivity coefficient. Indeed this is largely true for transitivity coefficient, but unexpectedly, clustering coefficient for NetIDP tends to be lower than that for NetAll (Table A in S1 File). However, when compared directly against degree-preserving randomizations of NetIDP, both coefficients are significantly greater than expected (Table 2 and Tables B and C in S1 File).

**Table 2:** Topological properties of NetAll and NetIDP for the four species at disorder threshold of 70%.

			NetIDP			
Properties	Species	NetAll	Measure	p-value	Mean	Std dev
**Network Density**	Yeast	0.0116	0.0141	-	-	-
	Drosophila	0.0042	0.0033	-	-	-
	Mouse	0.0038	0.0045	-	-	-
	Human	0.0024	0.0024	-	-	-
**Clustering Coeff**	Yeast	0.1622	0.0682	5.29E-195	0.0344	0.0079
	Drosophila	0.1561	0.0626	0.00E+00	0.0149	0.0038
	Mouse	0.2054	0.1362	0.00E+00	0.0309	0.0044
	Human	0.1957	0.0746	0.00E+00	0.0111	0.0032
**Transitivity Coeff**	Yeast	0.1071	0.0953	8.05E-201	0.0585	0.0082
	Drosophila	0.1360	0.1588	0.00E+00	0.0475	0.0082
	Mouse	0.11	0.1857	0.00E+00	0.0540	0.0058
	Human	0.0684	0.1473	0.00E+00	0.0338	0.0065
**Efficiency**	Yeast	0.4359	0.1599	-1.38E-125	0.1720	0.0039
	Drosophila	0.3277	0.0352	-4.72E-204	0.0541	0.0019
	Mouse	0.3452	0.0675	-0.00E+00	0.0946	0.0020
	Human	0.3612	0.039	-6.22E-257	0.0511	0.0017
**Charac Path length**	Yeast	2.4173	3.5271	5.84E-53	3.4714	0.0510
	Drosophila	3.2666	4.0255	1.65E-180	3.7837	0.0633
	Mouse	3.0592	4.3021	0.00E+00	3.5209	0.0416
	Human	2.9304	4.5982	3.68E-212	4.2151	0.0776
**Modularity**	Yeast	0.292	0.4901	4.63E-220	0.4572	0.0086
	Drosophila	0.3892	0.6111	3.1855e-314	0.5212	0.0082
	Mouse	0.4003	0.6476	0.00E+00	0.4593	0.0062
	Human	0.4154	0.7182	0.00E+00	0.5952	0.0067

The significance is assessed for each of the sub-networks based on degree preserving graph randomization whose mean and standard deviations are shown. The sign of the p-value indicates the directionality of the observed value relative to the mean of control values.

**Fig 1:**
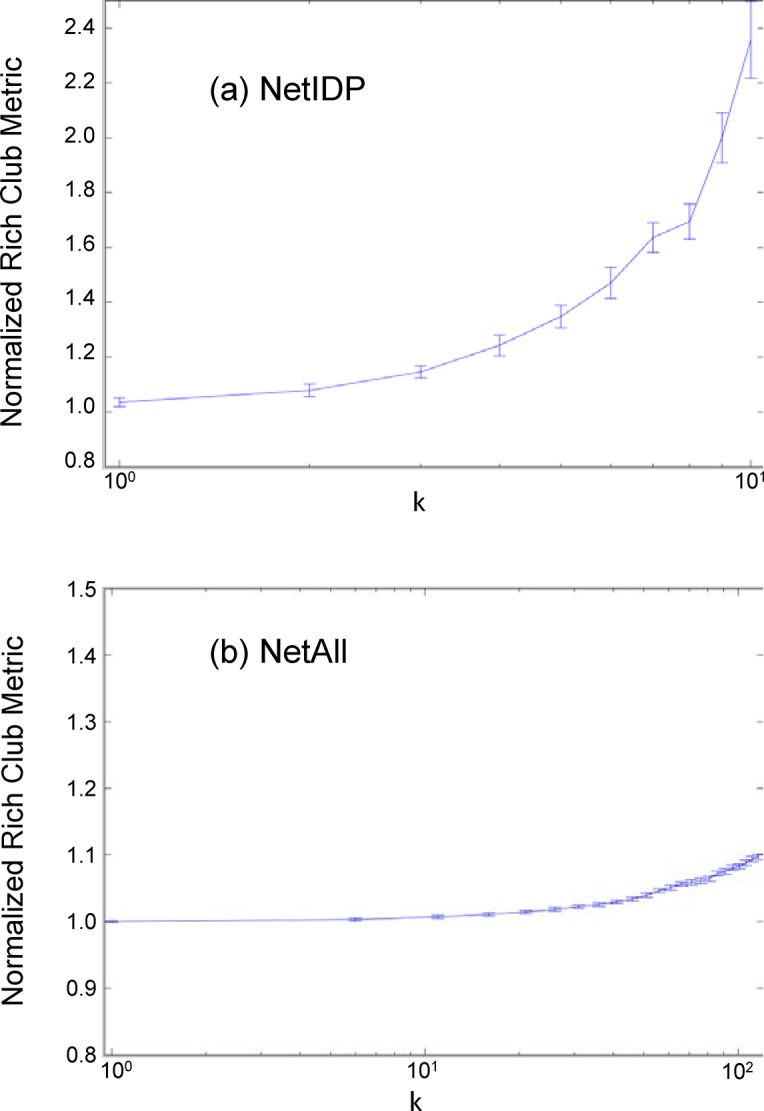
Normalized Rich club coefficient for NetIDP and NetAll for drosophila. The x-axis shows the degree threshold and y-axis shows the normalized rich club coefficient for nodes with a degree equal or greater to the threshold. Error bars are estimated based on bootstrapping over the nodes in the graph, and represent one standard deviation.

*Rich Club Coefficient* measures interconnectivity among nodes with degree above a certain threshold and is normalized against degree-preserving randomized graphs (Table 1). Normalized rich club coefficient for NetIDP, and NetAll for drosophila are shown in Fig 1. Plots for other species are qualitatively similar (the trend is strongest in yeast and drosophila) and not included. We found that across all species, NetIDP (but not NetAll) showed a rich club coefficient (values >1), which generally increases with increasing node-degree. Rich-club phenomenon represents a greater than expected inter-connectivity among large degree nodes, which is a crucial indicator of the presence of dominant communities [19].

### Modularity

Modularity quantifies how a network is organized into relatively independent communities. Modularity is estimated relative to a particular community structure, for which we used the approach described in [20], which maximizes the difference between intra-community edges to its expected value assuming random edge assignment. It is non-trivial to directly compare modularity of different graphs (NetIDP and NetAll in this case). Therefore using the community structure derived from NetAll, and by projecting IDPs onto that community structure, we estimated the modularity of NetIDP and compared it against its own degree-preserving randomized versions. Consistent with our expectations from the results above, we found that in in all cases (species, threshold, network source combination) without exception, NetIDP modularity was greater than expected (Table 2 and Tables B and C in S1 File). This suggests that highly disordered proteins are organized into modules.

We further investigated the distribution of IDPs among the NetAll-derived communities, and found that generally IDPs are uniformly distributed among the NetAll communities. We specifically tested whether each of the communities was enriched for IDPs using Fisher Exact test and found this not to be the case. For instance, in the human at 70% threshold, only 2 out of 23 communities are enriched for IDPs (p-value < 0.05) which is not significant after multiple testing correction; the same is generally true for all species and both thresholds.

### Characteristic Path Length and Efficiency

These are two related measures where the characteristic path length is the mean and efficiency is the harmonic mean of all pair-wise shortest paths. Efficiency relates to the physical efficiency of information exchange within a network [21]. A shorter path length generally implies greater efficiency. Note that these properties for NetIDP cannot be compared directly with those for the corresponding NetAll, because only the IDP nodes are considered for shortest path calculation. Instead, as for other properties, we assess the values for NetIDP relative to those for degree-preserving randomized NetIDPs. In our analysis, the two measures displayed a consistent trend. Out of 12 significance tests (4 species using STRING physical interaction network and human and yeast using BIOGRID, at two thresholds), in 11 cases, NetIDP’s characteristic path length is greater than expected and efficiency is lower than expected. A greater path length (correspondingly, low efficiency) in conjunction with greater-than-expected modularity may suggest that NetIDP consists of well-separated communities. This is consistent with a previous report that showed that, in social network context, modularity is inversely correlated with efficiency [22].

### Properties of IDP neighbors

Next we assessed the manner in which IDPs connect with the rest of the PIN. Toward this goal we first computed the betweenness centrality of every node and compared the values for three sets of nodes: (i) IDP nodes, (ii) non-IDP nodes connected to an IDP node, and (iii) the remaining nodes. As shown in Table 3 (and Table D in S1 File), we found that consistently, non-IDP nodes connecting the IDP nodes to the rest of the network have significantly higher betweenness centrality. This result supports a model where IDPs communicate with the rest of the network via an intermediate layer of critical, non-IDP mediators.

**Table 3:** Betweenness centrality of NetIDP, their neighbors and the rest of the non-neighbor nodes.

**Yeast**	
NetIDP	0.0003±0.0013
NetIDP Neighbors	0.0007±0.0067
Wilcoxon Test	neighbors' centrality > IDP centrality (p = 1.1075e-09)
NetIDP non-neighbors	0.00005±0.00012
Wilcoxon Test	neighbors' centrality > non-neighbors' centrality (p = 0)
**Drosophila**	
NetIDP	0.0004±0.0037
NetIDP Neighbors	0.0010±0.0048
Wilcoxon Test	neighbors' centrality > IDP centrality (p = 3.7346e-76)
NetIDP non-neighbors	0.00013±0.00032
Wilcoxon Test	neighbors' centrality > non-neighbors' centrality (p = 0)
**Mouse**	
NetIDP	0.0002±0.0015
NetIDP Neighbors	0.0007±0.0056
Wilcoxon Test	neighbors' centrality > IDP centrality (p = 2.0934e-66)
NetAll/NetIDP+	0.00006±0.00022
Wilcoxon Test	neighbors' centrality > non-neighbors' centrality (p = 0)
**Human**	
NetIDP	0.0001±0.0006
NetIDP Neighbors	0.0006±0.0138
Wilcoxon Test	neighbors' centrality > IDP centrality (p = 6.4789e-87)
NetAll/NetIDP+	0.00005±0.00023
NetIDP non-neighbors	neighbors' centrality > non-neighbors' centrality (p = 0)

Next, given that IDPs are organized into communities or modules, we assessed whether IDP neighbors exhibit a non-random association with the IDP modules. For each non-IDP node that is connected to at least one IDP, we determined the number of distinct modules that the IDPs connected to the non-IDP node belong to. We then compared the number of IDP modules for the IDP neighbors with the corresponding numbers for randomized NetIDPs where the nodes in each module were randomly swapped with nodes in other modules (i.e. module assignment was randomized while preserving the module sizes). As shown in Table 4 (and Table E in S1 File), without exception, IDP neighbors connect to fewer than expected IDP modules. This suggests that the modularity of IDPs extends in the way they are connected to their neighbors.

**Table 4:** Analysis of number of NetIDP modules connected to a NetIDP neighbor.

Species	Disorder (%)	Avg #connected modules	mean	Std Deviation	p-value
**Yeast**	70	2.1322	2.5945	0.0339	0.00E+00
	50	3.1019	3.6544	0.0426	0.00E+00
**Drosophila**	70	1.6148	2.3682	0.0495	0.00E+00
	50	2.2837	3.3397	0.0667	0.00E+00
**Mouse**	70	1.6507	2.5410	0.0658	0.00E+00
	50	2.0739	3.1360	0.0491	0.00E+00
**Human**	70	1.6830	2.2142	0.0364	0.00E+00
	50	2.0096	2.8535	0.0393	0.00E+00

The mean and standard deviation is estimated from 300 randomized module assignments of NetIDP (see text for details).

### IDPs interact preferentially with other IDPs

A previous report has found that IDPs preferentially interact with other IDPs [23]. We assessed the tendency of IDPs to assort with each other by comparing the observed ratio of IDP-IDP edges to IDP-non-IDP edges with the expected ratio (based on number of IDP and non-IDP nodes) via Fisher test. As shown in Table 5, in all cases, the tendency of IDPs to assort with other IDPs is observed.

**Table 5:** IDPs tend to preferentially interact with other IDPs.

Species	Disorder %	Observed ratio	Expected Ratio	Fisher test p-value
**Yeast**	70	0.0230	0.0188	3.83E-04
	50	0.0735	0.0495	0
**Drosophila**	70	0.0315	0.0279	0.0171
	50	0.1025	0.0799	0
**Mouse**	70	0.0278	0.0214	0
	50	0.0878	0.0562	0
**Human**	70	0.0273	0.0225	9.30E-05
	50	0.0716	0.0530	0

The observed ratio is for IDP-IDP edges and IDP-non-IDP edges. The expected ratio is based on number of IDP and non-IDP nodes.

### IDPs occupy higher levels in regulatory hierarchy

A previous analysis of regulatory networks suggests a regulatory hierarchy where the top-level ‘master’ regulators control the bottom-level proteins via a layer of so call mid-level managers. We tested whether IDPs are enriched in a particular level of hierarchy. We obtained previously inferred hierarchy of human regulatory genes [24]. Based on shortest path analysis we identified nodes at 5 levels, with 58, 2063, 778, 171, and 18 genes respectively. We found that 24% of the top level genes are IDPs compared with 8.1%, 8.9%, 9.4%, and 5.5% at the next 4 levels respectively. Thus, IDPs are significantly enriched at the top level of regulatory hierarchy relative to other levels combined (Fisher exact test p-value = 0.0012).

### Functional Enrichment Analysis

Using NIH’s DAVID tool [25], we assessed enriched functional terms among proteins in NetIDP relative to NetAll. As shown in various tables in S1 File overall, the enriched Gene Ontology (GO) terms in the four species recapitulate previous studies that have shown that IDPs are enriched for GO terms related to transcription, chromosomal organization, development, cell cycle regulation, etc. GO terms related to transcription and chromosomal organization are enriched in NetIDP in all four species in our analysis.

**Fig 2:**
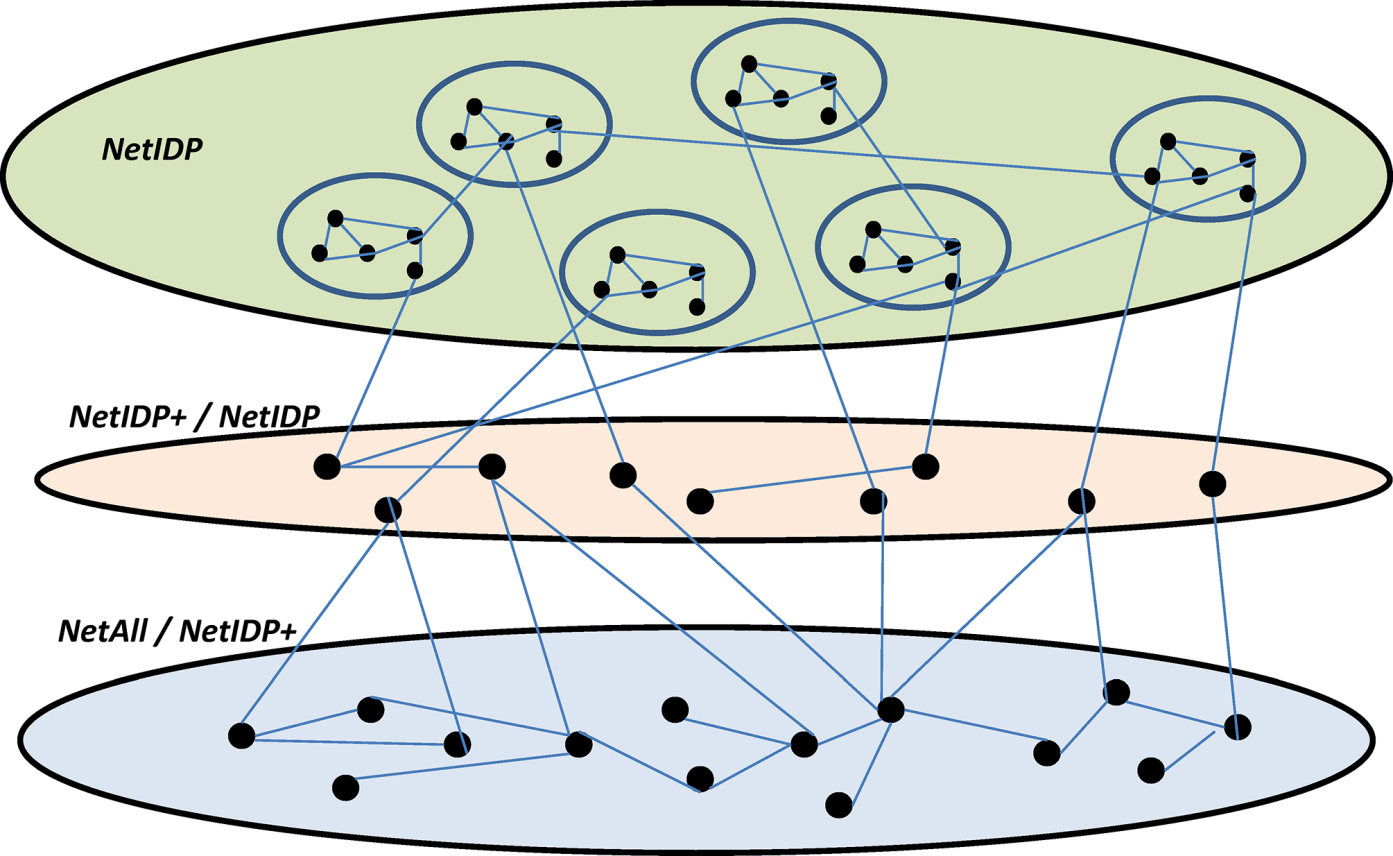
Proposed model. This figure illustrates the proposed model suggested by our comparative analysis of topological properties of IDP sub-networks. Our results are consistent with a network model wherein intrinsically disordered proteins (IDPs) are organized into a loose federation of relatively small tightly knit communities (topmost layer). The IDP layer is connected to the rest of the protein interaction network (bottom layer) via an intermediate layer (middle layer) of proteins, which have high betweenness centrality and connect to fewer than expected NetIDP modules.

Although functional properties of IDPs have been investigated before, the functions of the proteins that connect with IDPs and mediate the communication between IDPs and the rest of the PIN (middle layer in Fig 2) have not been investigated. We specifically assessed functional enrichment among non-IDP proteins that are directly connected to an IDP. Interestingly, we found that several functional terms were found enriched in NetIDP neighbors (at 70% threshold) in at least 3 of the 4 species but were not found enriched in NetIDP in any of the species. These are likely to represent unique functions enriched among structured proteins that interact with IDPs. These functional terms are listed in Table “GO-Unique-Nb” in S1 File. We found that in terms of molecular functions the IDP neighbors are uniquely enriched for DNA binding, kinase, helicase, ATPase activities, etc. In terms of biological processes these proteins are enriched for cell cycle, mRNA processing, translation, and transport.

### Osmotic stress response network in yeast

**Fig 3:**
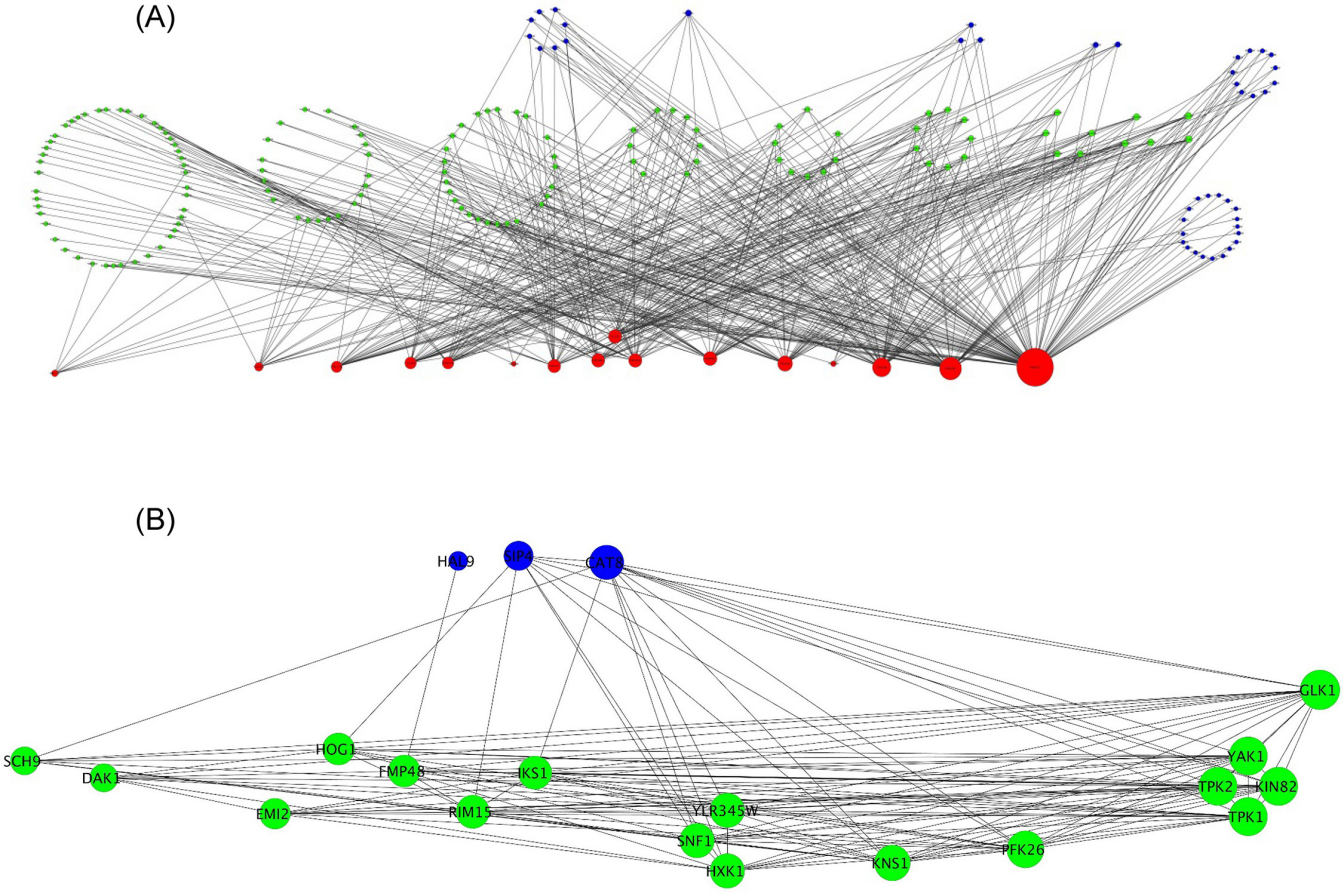
Yeast osmotic stress response network. **(A).** The figure shows the 17 IDP nodes involved in osmotic stress (red) as well as all other non-IDP nodes that are annotated as either ‘kinase’ (green) or ‘transcriptional regulator’ (blue) and connected to one of the 17 IDP nodes. Nodes are organized by the degrees. **(B)** Six of the 17 IDP genes (MSN2, HSP12, GRE1, SIP18, STF2, and USV1) were completely connected to each other. The figure shows 21 non-IDP genes annotated as ‘kinase’ (18 in green) or ‘transcriptional regulator’ (3 in blue) and connected to at least 4 of the 6 IDP genes. The 21 nodes and their connectivity are shown. Larger versions of the two figures are provided in additional files S2 and S3 Figs.

As an illustrative case study, we focused on *S*. *cerevisiae* because of its superior quality of functional annotation [26]. Osmotic stress response was one of the enriched biological processes uniquely enriched among yeast IDPs. Moreover, among the neighboring non-IDP nodes connected to IDP nodes, ‘kinase’ and ‘transcriptional regulator’ were two of the enriched terms. Fig 3A illustrates the network composed of the 17 IDPs involved in osmotic stress and all non-IDP nodes annotated to be either kinase or transcriptional regulators. To further clarify the osmotic stress network, out of 17 IDPs we identified a completely connected community of 6 genes: MSN2, HSP12, GRE1, SIP18, STF2, and USV1. We then identified 21 non-IDP nodes (18 kinases and 3 transcriptional regulators) that were connected to at least 4 of the 6 IDP nodes listed above. Fig 3B shows the 21 non-IDP nodes and their connectivity. For clarity we have excluded the IDP nodes because the 21 non-IDP nodes are densely connected with the 6 IDP nodes which form a clique among themselves. The majority of these neighboring nodes connected to the osmotic stress response genes are themselves involved in mediating various kinds of stresses including osmotic stress, hypoxia, starvation, heat, radiation, and replication stresses. This illustrative example suggests that a core IDP sub-network may coordinate the activities of several tightly linked kinases and transcriptional regulators to mediate the response to various stressors in yeast.

## Discussion

Networks of genes and proteins underlie the information processing capability of biological systems. Therefore, the architecture of PINs must critically determine the functional complexity, robustness, and evolvability of biological systems, thus motivating an investigation of their topological properties [5,6,7,8,27]. Due to their ability to interact with multiple partners [10,11], IDPs occupy central positions in PINs [9]. They are involved in key biological processes including development. Furthermore, perturbations of IDPs and IDP networks can lead to pathological states including cancer [28,29,30]. While the distinctive status of IDPs in PINs is well known, the broader topological properties induced by IDPs have not been investigated previously. Such topological properties could be important in understanding the broad functional role of IDPs in development and disease. Here, we conducted a comprehensive survey of the topological properties of IDP-induced sub-networks independently in four different species. However, we emphasize that the conservation of topological properties across species is not simply due to conserved networks, because the fraction of genes that have identifiable orthologs (www.ensembl.org) in each of the four species included here ranges from 5% to 32%.

Incompleteness and false positive of available PPI databases plagues all studies relying on large PPI networks. There are numerous PPI databases, each with overlapping focus and underlying philosophy, but ultimately, all of them relying on some form of literature-based curation and include high-throughput assays such as Yeast two hybrid (Y2H). While in the absence of a large ‘gold standard’ set, it is not clear how to tease out database specific biases, highly interacting and well-studied proteins are better represented in these databases. Based on our degree distribution comparison of IDPs and the rest of the proteins, high interaction is not likely to bias our analyses. We have used STRING *experimental track* for our main discussion, which integrates 7 independent PPI databases—BIND, DIP, GRID, HPRD, IntAct, MINT, and PID, and in doing so it is expected to implicitly minimize database-specific biases. And more generally, by analyzing multiple species, as there can be species-specific biases in database quality, and also a stand-alone database –BioGRID, not included in STRING, we tried to ensure that the trends we observe are robust.

We observe a greater edge density in NetIDP than NetAll, and although a greater-than-expected overlap between IDPs and hubs has been previously reported [9], this specific trend is not clearly recapitulated in our analysis. The correspondence between hubs and IDPs is likely to be weak, if at all, and as such there are important differences between ‘hubs’ and IDPs that are worth clarifying. While hubs, as a broad class, are evolutionarily conserved [31], IDPs are known to evolve under relaxed purifying selection [32,33]; however, there are examples of proteins where the disordered regions seems to be evolutionarily more conserved than the ordered regions [32,34,35]. Moreover, hubs tend not to interact directly with other hubs, whereas IDPs preferentially interact with other IDPs and this tendency is much stronger for non-hub IDPs [23]. This general tendency of IDPs to have higher connectivity and assortativity with other IDPs bears out in our analysis. In fact, our analysis further clarifies these previously observed tendencies by revealing a modular organization among IDPs, which is consistent with both greater connectivity and greater assortativity.

While IDPs commonly interact with multiple partners, there are instances where a structured protein, acting as a hub, interacts with multiple, disordered partners, *e*.*g*., the members of 14-3-3 protein family [36]. Interestingly, various 14-3-3 paralogs maintain a distinct set of interacting partners, and the average disorder of the partners of each 14-3-3 paralog was shown to correlate with the clustering coefficient of the sub-network composed of the paralog and the partners. This correlation between average disorder and clustering coefficient is expected due to greater tendency of IDPs to interact with other proteins. Interestingly, in our analysis, we found that NetIDP has a lower clustering coefficient relative to NetAll suggesting that IDPs are not connected with each other indiscriminately, but rather form a more structured modular architecture.

We found that relative to degree-preserving randomized graphs, NetIDP has greater-than-expected clustering, transitivity, and rich club coefficients. However, these are not a simple consequence of greater edge density since we also see a greater-than-expected modularity and characteristic path length in NetIDP. This may suggest that IDPs form smaller connected communities with loose connectivity across communities, thus increasing the overall characteristic path length in NetIDP.

Spurred by the finding that biological as well as many non-biological networks exhibit a power-law degree distribution [4], a lot of attention thus far has been on the highly connected nodes or hubs [37,38,39] as well as nodes with high betweenness centrality [40]. For instance, a centrality-lethality rule was posited based on the tendency of hub proteins to be essential [41,42]. However, later studies have further resolved hubs and placed particular importance on the so-called date hubs which are transiently connected to their partners and when deleted, have a much greater effect on network connectivity as they tend to connect distinct functional modules i.e., they have greater betweenness centrality [39,43,44]. Instead of ascribing a high betweenness centrality to IDPs, our analysis shows that the non-IDP nodes directly connected to IDPs have an even greater betweenness centrality. Moreover the non-IDP neighbors of IDPs connect to fewer-than-expected IDP modules. Together, these results may suggest that the IDP neighbors serve as entry-points mediating the communication between (a small set of) IDP modules and various pathways and processes.

Previous research suggests that hierarchical modularity may represent a robust and generic property of biological networks and that most functional classes appear as relatively segregated sub-networks within the hierarchy [8]. For instance, such a hierarchical structure has been previously posited for regulatory networks, where certain transcription factors assume the role of master regulators and affect maximum gene expression changes via a layer of middle level managers, which in turn communicate with regulatory proteins at the bottom level [16]. Even though, unlike the regulatory networks in [16], PINs we employ do not have directionality of information flow [16], our results show, for the first time, that IDPs are in fact significantly overrepresented among the top-level regulators. Taken together, our analysis suggests an IDP-centric organization where IDP modules, at higher levels of regulatory hierarchy, communicate with the rest of the PIN via a middle layer of critical proteins that facilitate communication between a small number of IDP modules and the rest of the PIN.

## Conclusion

Overall, we have reported the first detailed analyses of topological properties of IDP-induced sub-networks in four highly diverged species. Taken together, our results are consistent with a network model wherein IDPs are organized into communities that, from a higher level of regulatory hierarchy, communicate with the rest of the PIN via an intermediate layer of proteins with high betweenness centrality and which connect to smaller-than expected IDP modules. Although the observed trends are suggestive of a particular organizational structure, deeper evolutionary and functional insights into these intriguing, albeit preliminary, observations will require a more detailed analysis aided by directed experiments of specific biological pathways enriched in IDPs.

## Materials and Methods

### IDP Identification

MobiDB database [45] integrates multiple computational and experimental sources to provide a consensus annotation of disorder for each residue in a protein sequence. Thus, MobiDB annotations were obtained for each of the four species investigated. We defined disordered regions as stretches of at least 30 consecutive amino acids. As described previously, the overall disorder score for the entire protein sequence was calculated as the fraction of protein sequence covered by disordered regions [9,46]. A protein was designated as an IDP if the disorder score, i.e., fractional coverage of the protein with disordered residues, was above a certain threshold. We used two thresholds, namely, 50% and 70% disorder.

There are alternative databases for characterizing protein disorder. For instance, the D2P2 database [47] integrates multiple computational predictions into a consensus score at each residue of a protein. Overall the disorder scores calculated by MobiDB and D2P2 are highly correlated (overall correlation of 0.72 for the four species, and as high as 0.92 for fly). Given the overall consistency and the fact that MobiDB relies on experimental data in addition to computational predictions, we performed all our analyses using MobiDB annotations.

### Protein interaction networks

For each of the four species, we obtained the PINs from the STRING database [17] which integrates multiple sources of information to obtain high confidence interactions between protein pairs. For the primary analyses we only used the physical interaction ‘experimental’ track without any filtering (mainly to assure sufficient data for statistical robustness) but for secondary analyses we used the integrated STRING database with default setting. For human and *S*. *cerevisiae*, we additionally used BIOGRID [18] as an alternative source for the PINs. From the entire network (NetAll) of each species, we derived NetIDP at different IDP thresholds.

### Network properties

The network properties included in this study are defined in Table 1. The network properties were calculated using the Brain Connectivity Toolbox [48] implemented in Matlab [49].

### Estimating significance of differences in a network property

For a specific network property for NetIDP (say, clustering coefficient), to estimate its significance, we applied a degree-preserving graph randomization approach implemented in Matlab, where the randomized network has identical degree distribution as the actual network. Based on clustering coefficient values for 300 such randomized graphs, we calculated a p-value for the observed clustering coefficient. We first checked whether the values from control samples come from a normal distribution using Lilliefors test [50]. In the rare cases when they did not, we applied the Box-Cox transformation [51] to obtain a normal distribution. Once normality was assured, we applied the Student's t-test to obtain the required p-value. We decided to do only 300 randomizations for computational speed. However, we think it is sufficient given that in a vast majority of cases the distributions fit a normal distribution.

## Supporting Information

S1 Fig Includes the degree distributions. (PDF)

S2 Fig Includes the large version of Fig 3A. (JPG)

S3 Fig Includes the large version of Fig 3B. (JPG)

S1 File Includes multiple excel sheets. (XLS)
