# Supplementary material for: Evolutionarily Conserved Network Properties of Intrinsically Disordered Proteins
Source: PLoS One. 2015 May 14;10(5):e0126729. doi: 10.1371/journal.pone.0126729 (PMC4431869; doi:10.1371/journal.pone.0126729)
Supplement: S1 Fig — (PDF) [file pone.0126729.s001.pdf]

Degree distribution of all nodes and IDP nodes based on STRING physical interactions

(a) Yeast

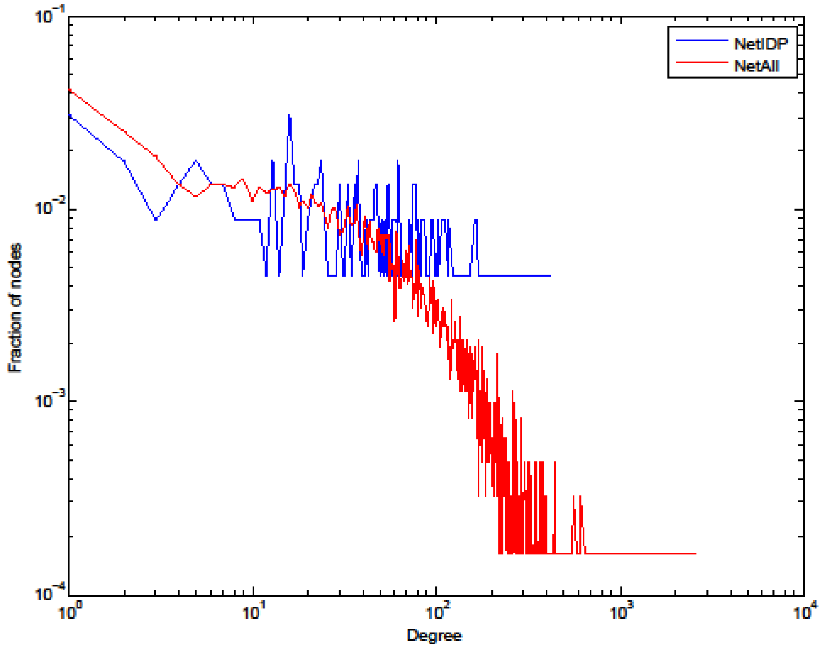

(b) Drosophila

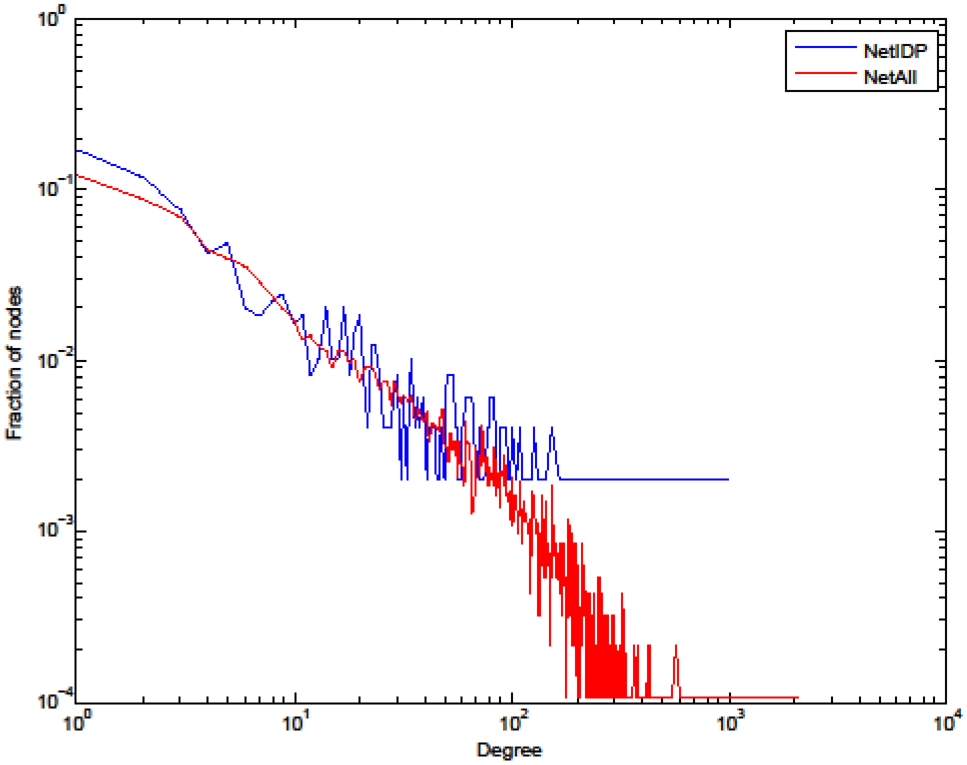

(c) Mouse

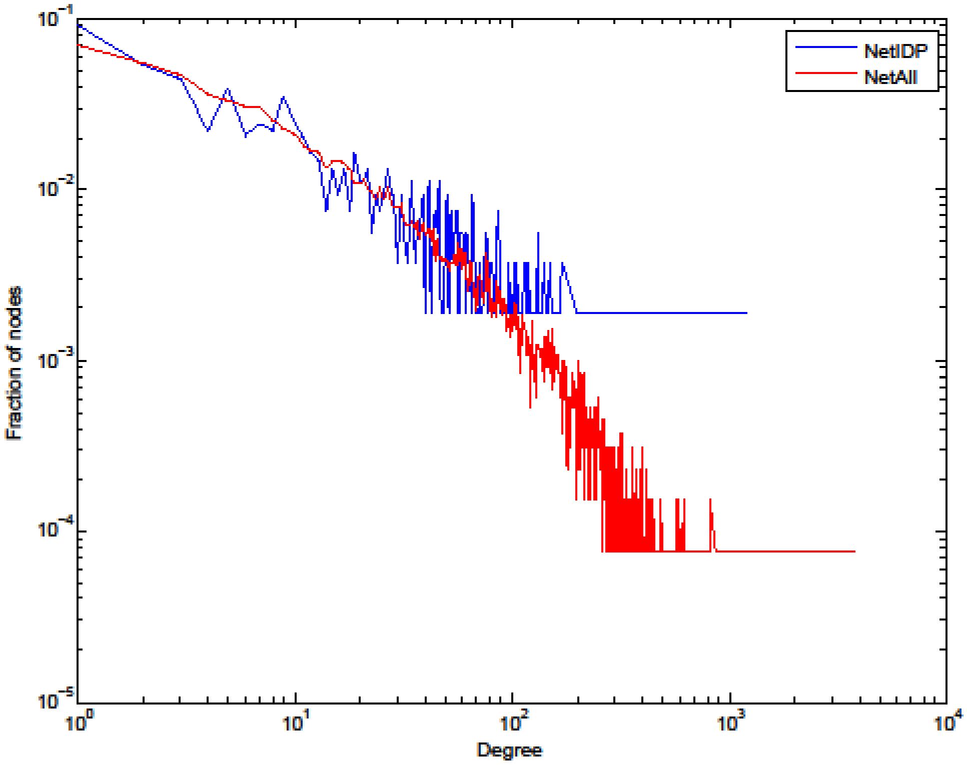

(d) Human

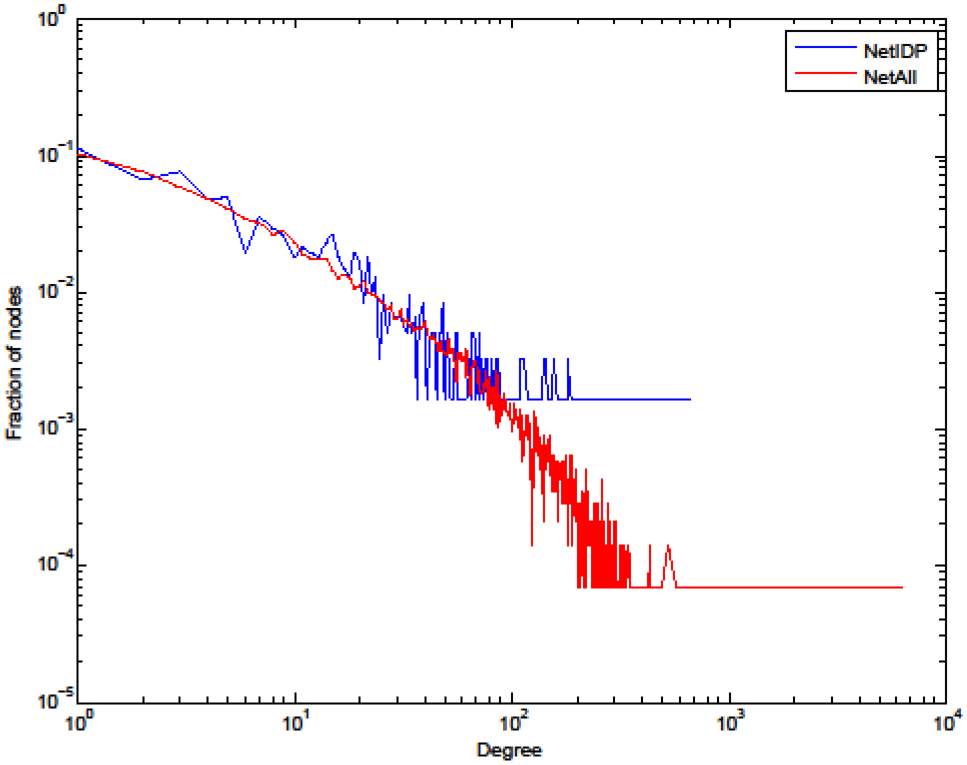
NetIDP
NetAll
Fraction of nodes
Degree
10^0
10^{-1}
10^{-2}
10^{-3}
10^{-4}
10^{-5}
10^0
10^1
10^2
10^3
10^4
